# RETRACTION: miR‐107 Regulates Growth and Metastasis of Gastric Cancer Cells via Activation of the PI3K‐AKT Signaling Pathway by Down‐Regulating FAT4

**DOI:** 10.1002/cam4.71599

**Published:** 2026-03-02

## Abstract

**RETRACTION**: WangL. , LiK. , WangC. , ShiX. , and YangH. , “miR‐107 Regulates Growth and Metastasis of Gastric Cancer Cells via Activation of the PI3K‐AKT Signaling Pathway by Down‐Regulating FAT4,” Cancer Medicine 8, no. 11 (2019): 5264–5273, 10.1002/cam4.2396.31297980 PMC6718591

The above article, published online on 12 July 2019 in Wiley Online Library (wileyonlinelibrary.com), has been retracted by the journal Editor‐in‐Chief, Stephen Tait; and John Wiley & Sons Ltd. Concerns were raised by third parties regarding several areas of overlap in Figures 3A and 3B; duplications in Figure 4; and irregularities with the blots in Figures 1C, 5AB, and 6AB. The authors were unable to provide satisfactory explanations or original data to resolve these concerns. These issues undermine the reliability of the data and the conclusions drawn from the study.

The authors have been informed about the retraction.

**RETRACTION**: L. Wang , K. Li , C. Wang , X. Shi , and H. Yang , “miR‐107 Regulates Growth and Metastasis of Gastric Cancer Cells via Activation of the PI3K‐AKT Signaling Pathway by Down‐Regulating FAT4,” Cancer Medicine 8, no. 11 (2019): 5264–5273, 10.1002/cam4.2396.31297980 PMC6718591

The above article, published online on 12 July 2019 in Wiley Online Library (wileyonlinelibrary.com), has been retracted by the journal Editor‐in‐Chief, Stephen Tait; and John Wiley & Sons Ltd. Concerns were raised by third parties regarding several areas of overlap in Figures 3A and 3B; duplications in Figure 4; and irregularities with the blots in Figures 1C, 5AB, and 6AB. The authors were unable to provide satisfactory explanations or original data to resolve these concerns. These issues undermine the reliability of the data and the conclusions drawn from the study.

The authors have been informed about the retraction.

